# Interactions between genes altered during cardiotoxicity and neurotoxicity in zebrafish revealed using induced network modules analysis

**DOI:** 10.1038/s41598-023-33145-8

**Published:** 2023-04-17

**Authors:** Manusmriti Agarwal, Ankush Sharma, Andrea Kagoo R., Anamika Bhargava

**Affiliations:** Department of Biotechnology, Indian Institute of Technology Hyderabad (IITH), Kandi, Telangana 502284 India

**Keywords:** Gene regulatory networks, Bioinformatics, Predictive markers, Modularity, Experimental models of disease

## Abstract

As the manufacturing and development of new synthetic compounds increase to keep pace with the expanding global demand, adverse health effects due to these compounds are emerging as critical public health concerns. Zebrafish have become a prominent model organism to study toxicology due to their genomic similarity to humans, optical clarity, well-defined developmental stages, short generation time, and cost-effective maintenance. It also provides a shorter time frame for in vivo toxicology evaluation compared to the mammalian experimental systems. Here, we used meta-analysis to examine the alteration in genes during cardiotoxicity and neurotoxicity in zebrafish, caused by chemical exposure of any kind. First, we searched the literature comprehensively for genes that are altered during neurotoxicity and cardiotoxicity followed by meta-analysis using ConsensusPathDB. Since constant communication between the heart and the brain is an important physiological phenomenon, we also analyzed interactions among genes altered simultaneously during cardiotoxicity and neurotoxicity using induced network modules analysis in ConsensusPathDB. We observed inflammation and regeneration as the major pathways involved in cardiotoxicity and neurotoxicity. A large number of intermediate genes and input genes anchored in these pathways are molecular regulators of cell cycle progression and cell death and are implicated in tumor manifestation. We propose potential predictive biomarkers for neurotoxicity and cardiotoxicity and the major pathways potentially implicated in the manifestation of a particular toxicity phenotype.

## Introduction

Zebrafish (*Danio rerio*) is a well-known vertebrate model organism to study toxicology^1,2^. Advantages like cost-effective maintenance, high fecundity, external fertilization, short life cycle, fast growth rate, well-defined developmental stages, transparency of zebrafish embryos and, small size of the embryos as well as adults have made it ideal for toxicity testing^3^. Zebrafish also shows remarkable physiological and functional similarities with humans^4^. Approximately 70% of human genes have functional orthologs in zebrafish^5^. The use of zebrafish larvae also brings direct benefits of the 3R’s (Replace, Reduce, Refine) principle^6,7^.

Owing to rising critical health concerns upon toxicant exposure, this study focuses on the impact of such chemicals on gene expression networks during neurotoxicity and cardiotoxicity in zebrafish. Zebrafish is already an established animal model for evaluating cardiotoxicity and neurotoxicity as most of the signalling pathways and genes are conserved. This study sought to leverage the knowledge gained from zebrafish to model the potential gene networks in humans that may be affected during cardiotoxicity and neurotoxicity. We used meta-analysis to examine the alteration in genes during cardiotoxicity and neurotoxicity in zebrafish, caused by chemical exposure of any kind, using the database—ConsensusPathDB. ConsensusPathDB is a database that integrates information from multiple databases (31 public databases and constantly being updated) about physical entities and functional interactions to provide a more complete picture of biological interactions at the cellular level^8,9^. It achieves this through the application of a merging algorithm that identifies overlaps among physical entities/molecules (genes, proteins, enzymes, protein complexes and RNA) and interactions separately to identify the number of entities and interactions that are unique to each database. Upon input of genes that are differentially expressed in a particular phenotype, induced network module analysis of ConsensusPathDB can be used to generate interaction networks. The analysis maps the submitted genes to physical entities across all databases and then integrates information on interactions involving the entities from these databases. Multiple sub-networks are generated as modular hubs within the larger network of the studied phenotype, which may be concerned with different pathways. Through information on intermediate nodes and the types of entities and interactions that mediate a particular phenotype, the induced networks of ConsensusPathDB help generate novel insights that can be explored experimentally^10^.

Neurotoxicity is the ability of a chemical, biological, or physical agent to cause adverse functional or structural changes in the nervous system^11^. Advantages like easy penetration of chemicals through the external chorion membrane of zebrafish larvae by passive diffusion, development of the blood–brain barrier similar to mammals, and fast brain development have made zebrafish ideal for neurotoxicity screening^12^. Neurotoxicity endpoints include gene expression patterns, neural morphogenesis, and neuro-behavioral profiling^13^. We considered altered expression of genes related to neuronal function as neurotoxicity endpoint. Cardiotoxicity is defined as the toxicity that damages the heart muscle and other cardiac tissues and/or disrupts the electrophysiology of the heart^14^. Zebrafish are particularly suited to access cardiotoxicity since the heart rate and action potential are analogous to humans^15^. Along with this, optically transparent embryos allow the use of non-invasive techniques and whole animal imaging for cardiotoxicity evaluation. Cardiac function assessment can also be carried out by investigating teratogenic effects and through a variety of hemodynamic parameters, including heartbeat, cardiac output, fractional area change, fractional shortening, and vascular blood flow velocities and altered gene expression^16^. For the analysis in this study, we considered altered cardiac gene expression as cardiotoxicity endpoint.

In this study, we first manually curated articles published between 2019 and 2020 related to neurotoxicity and cardiotoxicity. We then subjected the genes altered during neurotoxicity and cardiotoxicity to interaction analysis using ConsensusPathDB. Pathophysiological interplays between the nervous and cardiovascular systems prompted us to investigate if there were any common target genes between neurotoxicity and cardiotoxicity and if known interactions existed between them.

## Results

### Interaction between genes upregulated exclusively during neurotoxicity in zebrafish

**Figure 1:**
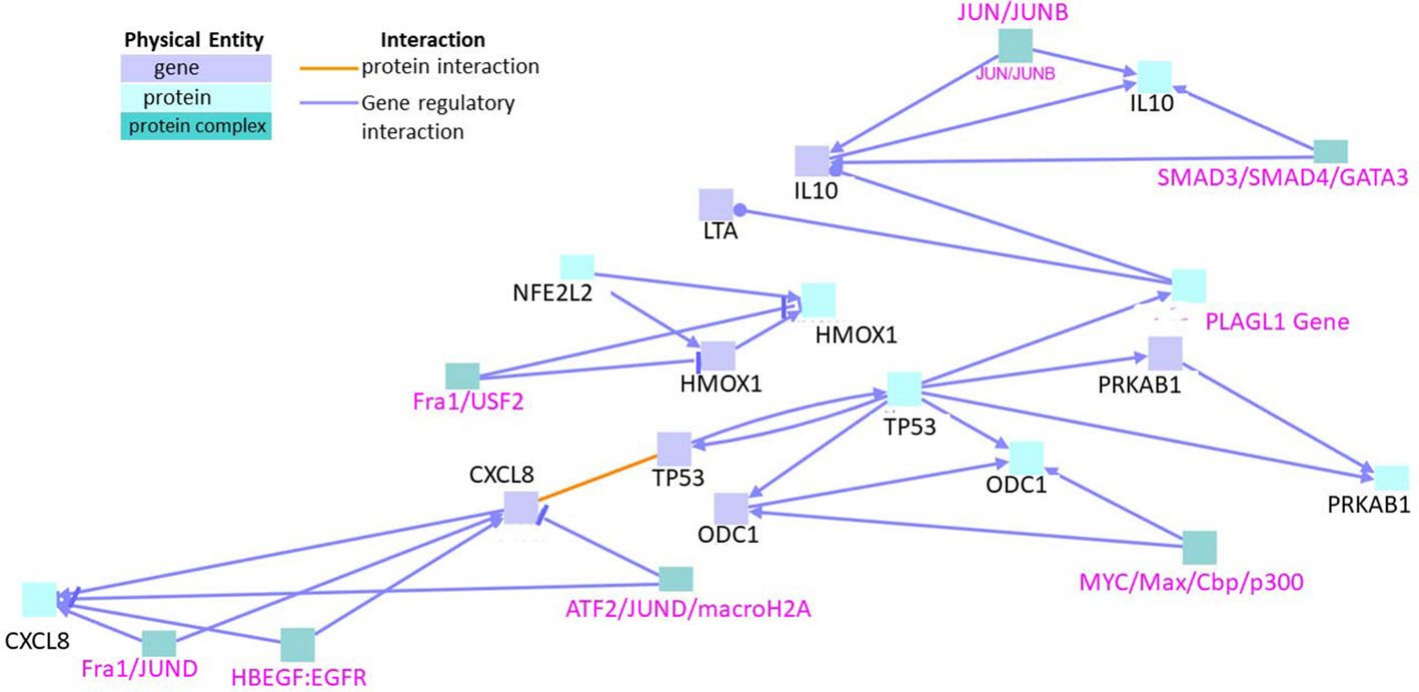
Interactions between genes that are exclusively upregulated in neurotoxicity. Black labels denote seed nodes and pink labels denote intermediate nodes. Each line represents an interaction. Line ending with an arrow represents stimulation whereas line ending with a small blunt line represents inhibition.

First, we analyzed the interactions between genes that were exclusively upregulated during neurotoxicity, excluding genes that were upregulated during both neurotoxicity and cardiotoxicity. This list of genes was subjected to “induced network modules analysis” in the gene set analysis in ConsensusPathDB. The analysis yielded two clusters (Fig. 1). The first cluster comprised seed node IL10 (Interleukin-10) (annotated in black) which is a cytokine with potent immunosuppressive potential^17^. Intermediate nodes (annotated in pink) included transcription factors such as SMAD3/SMAD4/GATA3 and JUN/JUNB. This cluster also included gene regulatory interactions mediated by LTA (Lymphotoxin alpha), TP53 (tumor suppressor p53), PRKAB1 (Protein Kinase AMP-Activated Non-Catalytic Subunit Beta 1), ODC1 (ornithine decarboxylase), and CXCL8 (C-X-C Motif Chemokine Ligand 8 also known as Interleukin-8). A sub-cluster with seed node LTA and an intermediate node protein encoded by the PLAGL1 (Pleiomorphic Adenoma Gene-Like 1) gene connected hubs centered on IL-10 and TP53. LTA is known to mediate a large variety of inflammatory and immunostimulatory responses^18^. Another sub-cluster was formed by seed nodes like PRKAB1, TP53, ODC1, and intermediate node MYC/Max/Cbp/p300. Here, TP53 was central to the network and formed forward gene regulatory networks with both PRKAB1 and ODC1 genes and proteins, where additional intermediate node MYC/Max/Cbp/p300 was found to be involved in gene regulatory interactions specifically with ODC1. CXCL8 gene further displayed gene regulatory interactions with intermediate nodes HB-EGF/EGFR, ATF2/JUND/macroH2A, and Fra1/JUND. The ATF2/JUND/macroH2A gene regulatory interaction was interestingly shown to have an inhibitory effect on the CXCL8 gene and protein. CXCL8 is a major mediator of the inflammatory response^19^. p53 is also known to be involved in the regulation of several proinflammatory genes in human macrophages like IL-6, IL-8, and CXCL1 (C-X-C motif chemokine Ligand 1)^20^. This confirmed the importance of the seed nodes in this cluster as major inflammatory contributors to neurotoxicity.

The second cluster consisted of seed nodes NFE2L2 (Nuclear Factor, Erythroid 2 Like 2) and HMOX1 (Heme oxygenase) where the HMOX1 gene and protein were shown to be negatively regulated (inhibited) by the intermediate node Fra1/USF2. NFE2L2 is a transcriptional activator that binds to the ARE (antioxidant response elements) and protects against oxidative stress by upregulating other antioxidant proteins^21^. HMOX1 is involved in heme catabolism, it cleaves heme to form biliverdin. Biliverdin is subsequently converted to bilirubin and carbon monoxide (a gaseous neurotransmitter) by biliverdin reductase^22,23^. This gives it an anti-inflammatory property by upregulating IL10. It also exhibits cytoprotective effects since excess of free heme sensitizes cells to undergo apoptosis^24^. Overall, the interaction modules in this gene set indicated that most of the genes were linked with neuroinflammation.

Furthermore, a protein interaction was found between the TP53 gene and the CXCL8 gene. No further biochemical reaction or genetic interactions were observed between the nodes.

### Interactions between genes upregulated in both neurotoxicity and cardiotoxicity in zebrafish

**Figure 2:**
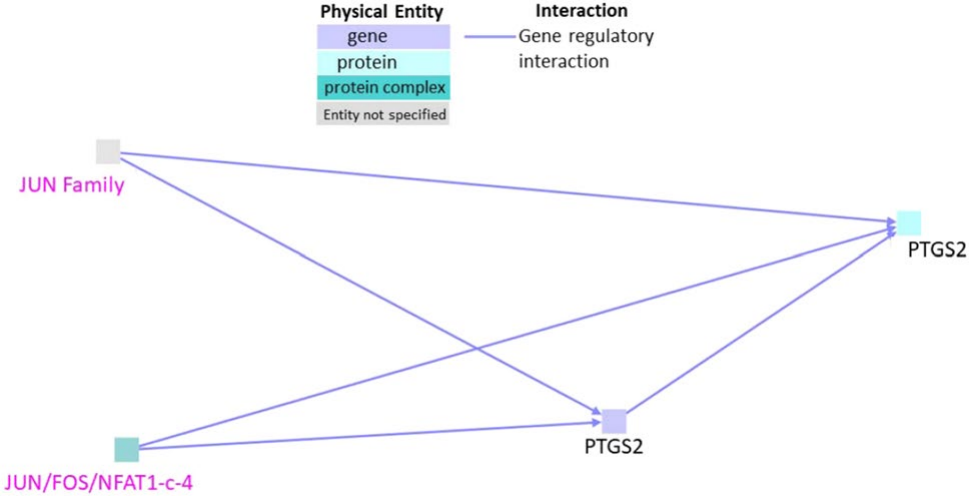
Interactions between genes that are upregulated both in neurotoxicity and cardiotoxicity. Black labels denote seed nodes and pink labels denote intermediate nodes. Each line represents an interaction. Grey square (JUN family) indicates that the entity whether gene/protein/compound etc. was not specified by the database.

When we subjected the list comprising genes that were upregulated in both neurotoxicity and cardiotoxicity to analysis in ConsensusPathDB, one cluster comprising only gene regulatory interactions was obtained (Fig. 2). Seed node PTGS2 (Prostaglandin G/H synthase 2 precursor) gene and protein were found to be involved in gene regulatory interactions that also involved two intermediate nodes namely JUN/FOS/NFAT1-c-4 and JUN family. FOS and Jun are transcription factors also linked to inflammation^25^. No further biochemical reaction, genetic, or protein interactions were observed between the nodes.

### Interactions between genes exclusively downregulated in neurotoxicity in zebrafish

**Figure 3:**
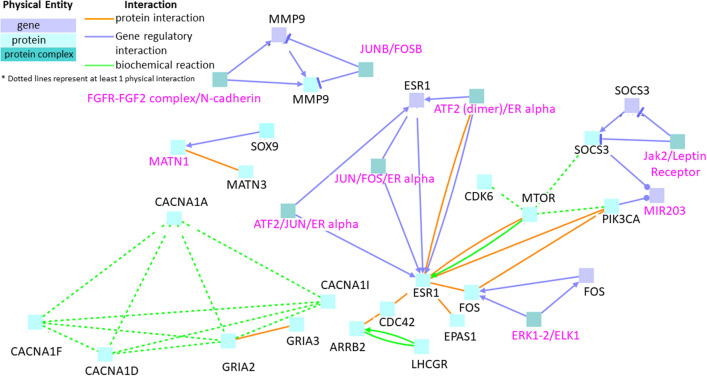
Interactions between genes that are exclusively downregulated in neurotoxicity. Black labels denote seed nodes and pink labels denote intermediate nodes. Each line represents an interaction. Dotted lines represent at least one physical interaction. Line ending with an arrow represents stimulation whereas line ending with a small blunt line represents inhibition.

When the list of genes downregulated in neurotoxicity excluding the commonly downregulated genes in neurotoxicity and cardiotoxicity, was subjected to the induced network modules analysis, four distinct clusters were obtained, three of which were anchored by gene regulatory interactions (Fig. 3). The first cluster comprised of three gene regulatory sub-clusters anchored by the seed genes FOS, ESR1 (Estrogen receptor alpha), and SOCS3 (Suppressor of cytokine signaling 3). The SOCS3 sub-cluster presented a complex network with ESR1 through protein and biochemical level interactions mediated by seed nodes PIK3CA (Phosphatidylinositol 3-kinase catalytic subunit, alpha isoform), and mTOR (Mammalian target of rapamycin) proteins. The SOCS3 sub-cluster elucidated the presence of intermediate nodes Jak2/Leptin Receptor and MIR203 through gene regulatory interactions. MTOR protein networked with ESR1 protein through biochemical as well as protein level interactions. Seed node proteins ARRB2 (Beta-Arrestin 2, isoform 1), CDC42 (Cell Division Cycle 42), PIK3CA, EPAS1 (Endothelial PAS domain-containing protein 1), and ESR1 interacted at the protein level. Further, ATF2/JUN/ER alpha, ATF2(dimer)/ER alpha, and JUN/FOS/ER alpha intermediate node pathways were implicated in the gene regulatory hub anchored in the ESR1 seed node, whereas ERK1-2/ELK1 was the intermediate node implicated in the sub-cluster anchored by the seed node FOS.

The second cluster comprised of seed node proteins SOX9 (SRY-Box Transcription Factor 9) and MATN3 (Matrilin 3) interconnected by an intermediate node protein MATN1 (Matrilin 1) through gene regulatory and protein level interaction, respectively. The SOX9 protein has been associated with initiation as well as progression of solid tumors^26^. The third cluster consisted of seed node MMP9 (Matrix metalloproteinase-9) and intermediate nodes JUNB/FOSB, Fra1/FIAT, and FGFR-FGF2 complex/N-cadherin.

When biochemical reaction interactions were included in the analysis, all the clusters interacted. Seed nodes like MTOR and CDK6 showed a physical biochemical reaction interaction (dotted green lines in Fig. 3). ARRB2 and LHCGR (luteinizing hormone/choriogonadotropin receptor) also showed networking at the biochemical level in the first cluster. The protein encoded by the mTOR gene belongs to a family of phosphatidylinositol kinase-related kinases. These kinases mediate cellular responses to stress such as DNA damage and nutrient deprivation^27^. This protein acts as the target for cell-cycle arrest and immunosuppressive effects of the (FK506 Binding Protein 12) FKBP12-rapamycin complex^28^. The protein encoded by the CDK6 gene is a member of the cyclin-dependent protein kinase family and is an important regulator of cell cycle progression^29,30^. They are known to be over-expressed in leukemia and malignancies^31^. ARRB2 is a major regulator of GPCR (G-Protein Couple Receptor) signaling including GPCR desensitization and internalization^32,33^. It also links GPCRs to clathrin-coated pits, regulation of cytoskeletal rearrangement and cellular localization, translocation, and regulation of signaling elements in the GPCR cascade^34^. The fourth cluster was devoid of any gene regulatory interactions and only showed proteins CACNA1A, CACNA1D, CACNA1I, CACNA1F, and GRIA2 (Glutamate ionotropic receptor AMPA type subunit 2) interacting via physical biochemical interactions. One can note that this group is comprised of mainly membrane receptors such as GPCRs and voltage-gated calcium channels like CACNA1D, CACNA1F, CACNA1A, and CACNA1I which may be associated physically in membrane microdomains.

Several protein–protein interactions were also observed in the proteins encoded by genes that were downregulated during neurotoxicity (orange lines in Fig. 3). The protein level interactions in the biggest cluster involved seed node proteins ARRB2, CDC42, ESR1, EPAS1, FOS (Fos Proto-Oncogene, AP-1 Transcription Factor Subunit), MTOR, and PIK3CA. EPAS1 also known as Hypoxia-inducible factor 2-alpha is a transcription factor that is induced when oxygen levels fall^35^. The fourth cluster, the only one devoid of any gene regulatory interactions implicated GRIA2 and GRIA3 in protein–protein interaction. GRIA3 plays an important role in excitatory synaptic transmission and is involved in bipolar disorder and nonspecific X-linked mental retardation^36^.

### Interactions between genes downregulated during neurotoxicity and cardiotoxicity in zebrafish

**Figure 4:**
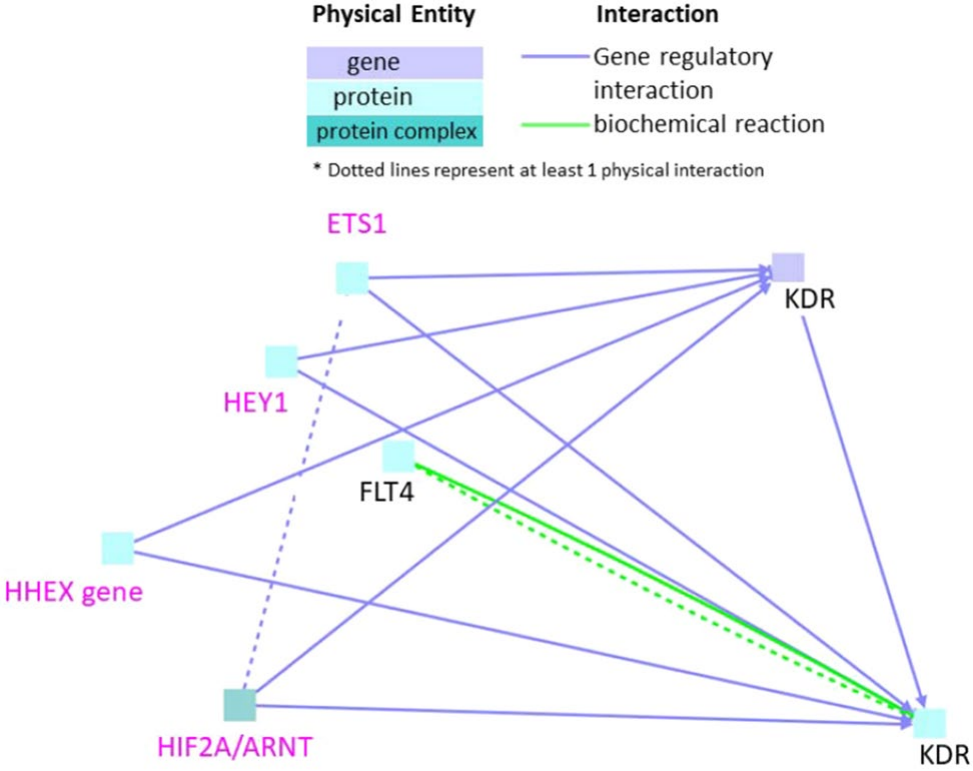
Interactions between genes that are downregulated in both neurotoxicity and cardiotoxicity. Black labels denote seed nodes and pink labels denote intermediate nodes. Each line represents an interaction. Dotted line represents at least one physical interaction.

Only two genes FLT4 (Fms-related tyrosine kinase 4) and KDR (Kinase Insert Domain Receptor) were found to be downregulated with a fold change of more than 3 in both neurotoxicity and cardiotoxicity. When these two genes were subjected to gene regulatory interaction analysis, few intermediate nodes were observed (Fig. 4). Intermediate nodes comprised of HIF2A/ARNT, HHEX gene, HEY1, and ETS1. KDR or VEGFR2 (Vascular Endothelial Growth Factor Receptor 2), which is a tyrosine-protein kinase acts as a cell surface receptor for VEGFA, VEGFC and VEGFD. It is known as a critical player in angiogenesis, vascular development and permeability^37^.

Seed nodes FLT4 and KDR interacted via biochemical reaction interactions (Fig. 4). FLT4 is a receptor tyrosine kinase for VEGFC and VEGFC. It is involved in adult lymph angiogenesis besides development of the cardiovascular system and vascular network during embryonic development^38^.

### Interactions between genes upregulated exclusively during cardiotoxicity in zebrafish

**Figure 5:**
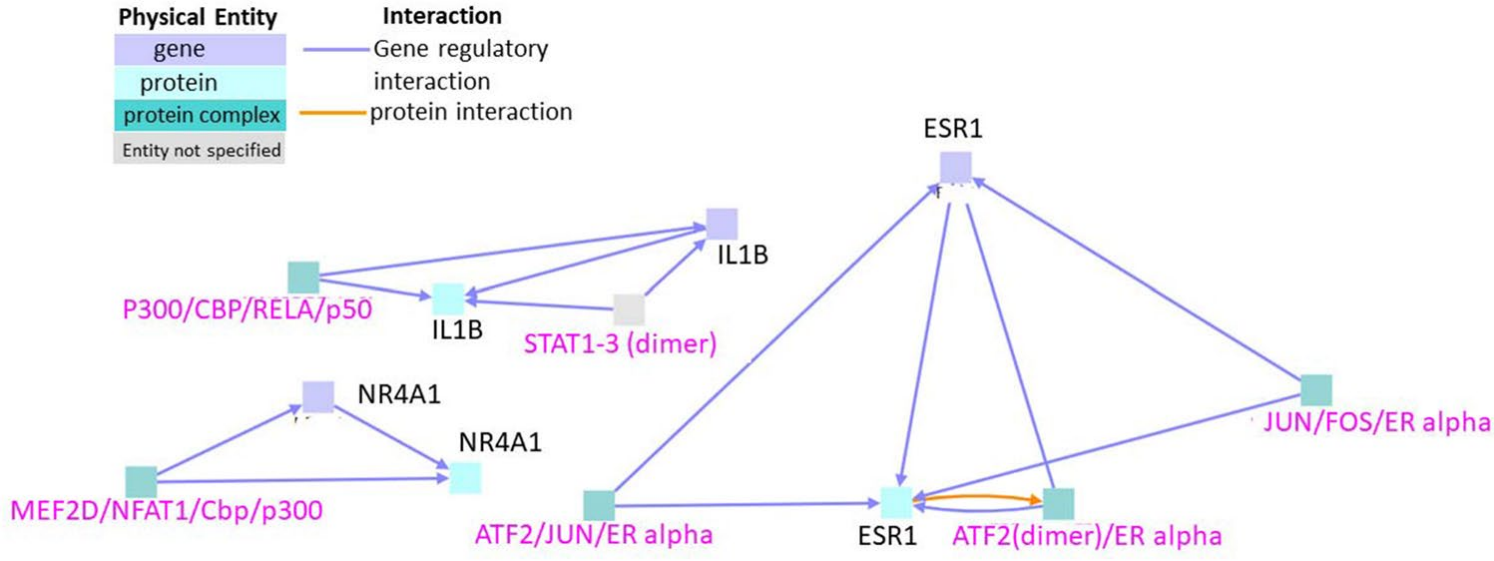
Interactions between genes that are upregulated exclusively in cardiotoxicity. Black labels denote seed nodes and pink labels denote intermediate nodes. Each line represents an interaction. Grey square (STAT1-3 dimer) indicates that the entity whether gene/protein/compound etc. was not specified by the database.

When genes that were upregulated during cardiotoxicity were subjected to gene regulatory interactions, three interaction clusters were observed (Fig. 5). Each of the three seed nodes ESR1, NR4A1 (Nuclear Receptor 4A subunit 1), and IL1β (Inerleukin-1 beta) formed a separate cluster. The protein encoded by NR4A1 acts as a nuclear transcription factor. This is an orphan nuclear receptor, the loss of which has been shown to lead to an inflammatory phenotype and increased atherosclerosis^39^. The protein encoded by the IL1β gene is a member of the interleukin 1 cytokine family, the major group of ligands and receptors associated with acute and chronic inflammation that play a central role in many cardiovascular diseases^40^. Suppression of IL1β has been used for treating severe symptoms in these conditions^40^. Intermediate nodes like JUN/FOS/ER alpha, ATF2 (dimer)/ER alpha, and ATF2/JUN/ER alpha were included in the first cluster. Intermediate nodes like MEF2D/NFAT1/Cbp/p300 were included in the second cluster. Intermediate nodes like STAT1-3 (Signal Transducers and Activators of Transcription) dimer and p300/CBP/RELA/p50 were included in the third cluster.

### Interactions between genes exclusively downregulated in cardiotoxicity in zebrafish

**Figure 6:**
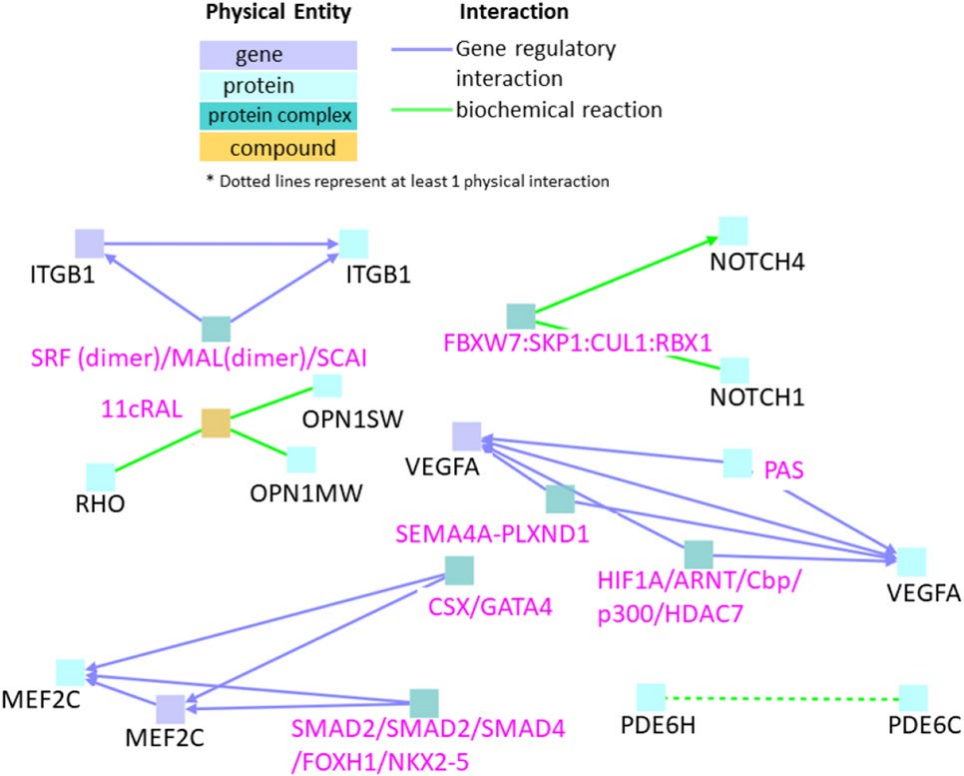
Interactions between genes that are downregulated exclusively in cardiotoxicity. Black labels denote seed nodes and pink labels denote intermediate nodes. Each line represents an interaction. Dotted line represents at least one physical interaction.

Three clusters of gene regulatory interactions were observed between the genes downregulated during cardiotoxicity (Fig. 6). Seed node VEGFA interacted with intermediate nodes PAS, HIF1A/ARNT/Cbp/p300/HDAC7, and SEMA4A: PLXND1 (Fig. 6). VEGFA (Vascular Endothelial Growth Factor A) is a growth factor that induces proliferation and migration of vascular endothelial cells^41^. In the second cluster, seed node ITGB1 (Integrin Subunit Beta 1) interacted with the intermediate node SRF (dimer)/MAL (dimer)/SCAI. ITGB1 are membrane receptors involved in cell adhesion, embryogenesis, homeostasis, tissue repair, immune response, and metastatic diffusion of tumor cells^42^. It has also been shown to increase stem cell survival and cardiac function after myocardial infarction^43^. In the third cluster, seed node MEF2C (Myocyte Enhancer Factor 2C) interacted with intermediate nodes SMAD2/SMAD2/SMAD4/FOXH1/NKX2-5 and CSX/GATA4. The protein encoded by MEF2C is a cardiac transcription factor that has been well-studied as a differentiation factor known to regulate a variety of cellular processes^44^.

Two additional clusters were observed with biochemical interactions. (Fig. 6). The first cluster consisted of seed nodes NOTCH4 and NOTCH1 (neurogenic locus notch homolog protein 4 and 1) and intermediate node FBXW7:SKP1:CUL1:RBX1. Mutations in the NOTCH1 gene have been implicated in multiple conditions including aortic valve disease, Adams-Oliver syndrome and leukemia, while mutations in the NOTCH4 gene have shown association with schizophrenia^45^. The second cluster consisted of seed nodes RHO (Rhodopsin), OPN1MW (Opsin 1, Medium Wave Sensitive, and OPN1SW (Opsin 1, Short Wave Sensitive) all of which interacted with a compound 11cRAL (11-cis-retinyl-Green-sensitive opsin, Opsin-1) that emerged as an intermediate node (Fig. 6 dark yellow square). The RHO gene encodes a protein found in rod cells; mutations in this gene are a cause of congenital blindness^46^. OPN1MW encodes opsins that are sensitive to medium-wavelengths of light while OPN1SW encodes for the blue cone pigment protein. Deuteranopic color blindness has been attributed to defects in OPN1MW gene, while tritanopia is attribute to mutations in OPN1SW^47,48^.

## Discussion

This study aimed at using gene expression data of cardiotoxicity and neurotoxicity phenotypes from the zebrafish animal model to understand similar gene networks in humans. Induced network modules analysis allowed to find major pathways involved in the phenotypes and to identify hidden molecular players that could be explored experimentally as useful biomarkers. Intermediate nodes thus obtained provided an opportunity to observe converging pathways and identify molecular players that may be affected, though not transcriptionally. These nodes may encode proteins that signal together in a microdomain complex and thus may be relevant for the particular phenotype^9^.

The interaction networks obtained in our study predominantly involved gene-regulatory interactions. The analysis was successful in identifying major pathways anchored by seed nodes (input genes) responsible for cardiotoxicity or neurotoxicity phenotype. Table 1 describes these anchoring seeds at the gene regulatory, protein as well as biochemical reaction levels. We propose that these genes and proteins possess the potential to be utilized as biomarkers for investigatingneurotoxicity and cardiotoxicity phenotypes in toxicity studies and for the assessment of health and diseased states in the zebrafish model organism and possibly in humans.

**Table 1:** Specific genes that can serve as biomarkers for studying toxicity in zebrafish (*Danio rerio*).

Interaction level	NT ↑	NT ↑ CT ↑	NT↓	NT↓ CT ↓	CT ↑	CT ↓
Gene regulatory	ODC1, PRKAB1, IL-10, TP53, LTA, NFE2L2, HMOX1	PTGS2	SOCS3, ESR1, MMP9, SOX9, FOS, PIK3CA	KDR	ESR1, IL1B, NR4A1	VEGFA, MEF2C, ITGB1
Protein	TP53-CXCL8		ARRB2, CDC42, PK3CA, ESR1, MTOR, GRIA-3, GRIA2, MATN3		ESR1	
Biochemical reaction			PIK3CA, CDK6, CACNA, MTOR, ESR1, LHGCR, ARRB2, SOCS3,	KDR, FLT4		NOTCH 1–4, RHO, OPN1SW, OPN1MW, PDE6H, PDE6C

Genes exclusively upregulated in Neurotoxicity (NT ↑); Genes upregulated in both Neurotoxicity and Cardiotoxicity (NT ↑ CT ↑); Genes exclusively downregulated in Neurotoxicity (NT↓); Genes downregulated in both Neurotoxicity and Cardiotoxicity (NT↓ CT ↓); Genes exclusively upregulated in Cardiotoxicity (CT ↑); Genes exclusively downregulated in Cardiotoxicity (CT ↓).

Estrogen receptor alpha (ESR1), a gene downregulated in neurotoxicity but upregulated in cardiotoxicity, revealed itself as a unique gene implicated in both the pathologies at both the gene-regulatory and protein level interaction. The ESR1 gene is generally implicated in estrogen resistance in breast cancer, it encodes for a nuclear hormone receptor and regulates cellular proliferation, and it also associates with DNA-binding transcription factors such as ATF-2, FOS, and JUN to mediate signaling independent of the estrogen response element^49,50^. These are also the intermediate nodes observed in our analysis. ESR1 has been shown to mediate neurodevelopmental toxicity in zebrafish upon exposure to aromatase and phthalates through impaired neurogenesis^51^. It also accelerates heart regeneration via inflammatory signaling^52^. These studies assert the results of the interaction modules analysis in our study. These cross-observations also indicate the potential of ESR1 to be employed as a biomarker for both phenotypes through pathways implicated in regeneration and inflammation. PTGS2, a key enzyme in prostaglandin biosynthesis, implicated in epilepsy^53^, cardiomyopathy and regeneration^54^ was also observed to be upregulated in both cardiotoxicity and neurotoxicity in our studies and through the interaction networks it associated with JUN family of transcriptions factors underscoring the overlapping pathways in toxicity and cancer. Similarly, the Kinase insert domain (KDR) gene, encoding for the VEGF receptor has been implicated in angiogenesis and glioma^55–57^ and was found to be downregulated in both neurotoxicity and cardiotoxicity phenotypes. These observations highlight a major use case of molecular players like ESR1, PTGS2, and KDR as biomarkers of the cardiotoxicity and neurotoxicity phenotypes anchored in regenerative and inflammatory signaling pathways. A unique observation of our study is the substantial overlap of genes involved in tumor manifestation/association with the genes involved in cardiotoxicity and neurotoxicity such as ESR1, PTGS2, LTA and KDR. Many of these genes are already proposed as cancer biomarkers^58–61^. It would be pertinent to determine if cardiotoxicity and neurotoxicity would lead to carcinogenesis though some epidemiological evidence exits. It is worth to mention that though there appears a convergence of toxicity pathways with cancer pathways, the intermediate nodes that group these networks into sub-networks underscore the absence of bias in our analysis and may represent a true overlap of toxicity pathways with cancer pathways.

The induced networks aim to connect as many input seed nodes as possible, however, certain seed nodes that displayed high differential expression in experiments, did not appear in the networks generated through ConsensuPathDB in our study. This absence however does not diminish the significance of genes such as cyp1a1, gstp1, cyp19A1, and Rgr. Some of these genes such as cyp1a1 and gstp1 are already associated with toxicity and may act as biomarkers for toxicity^62–64^. It is possible that they are involved in the toxicity manifestation through other functional interactions in differently connected pathways, and therefore are not depicted in the pathways in our figures.

In our study, input genes were not separated based on the differential expression at a particular developmental or adult stage. Most of the studies that are referenced, used embryonic/larval stages of zebrafish for toxicity analysis (Supplementary Table S9). Hence, our study may provide a limited opportunity to understand the concerned phenotypes from the perspective of adult zebrafish stages. However, all the genes used in our analysis are expressed in humans and therefore our results may be extrapolated to human toxicity.

Insights regarding the role and networking of these genes can serve to establish phenotypes and test hypotheses involving the novel intermediate genes revealed as a result of the induced network modules analysis. This study makes a case for utilizing gene expression data sets and induced network modules analysis to lead researchers to novel biomarkers that can be used to investigate and establish phenotypes.

## Methods

We searched the literature in academic databases PubMed and Google Scholar for articles that reported cardiotoxicity and neurotoxicity in zebrafish in the year 2019 and 2020. The study was conceptualised in early 2021 and hence literature from the previous two years was reviewed. Also, there was a considerable increase in the publications in this area from previous years and therefore it was not possible to sample all the publications. Therefore, we chose 2 years which had around 2678 publications with the search terms used in our study. The search terms used were—“Neurotoxicity in zebrafish”, “Cardiotoxicity in zebrafish”, “acute toxicity in zebrafish”, “Chronic toxicity in zebrafish”. 592, 218, 938 and 930 hits were received for each string, respectively. Then, the articles were manually screened for gene expression studies and data such as gene name, gene alteration (upregulated or downregulated), primer sequences, study design including the chemical used, time of exposure, the time point at which qPCR was done, strain, and the developmental stage of the zebrafish. Toxicity articles that did not report gene expression data were excluded from our analysis.

A total of 76 articles for neurotoxicity and 34 articles for cardiotoxicity were found relevant and were included in the analysis presented here (Titles of these articles are provided in the Supplementary Tables S1 and S2 respectively). From the data collected from the relevant articles, the gene lists were created and divided based on the observed alteration. Six gene lists were created: (1) Exclusively upregulated genes in neurotoxicity (Supplementary Table S3), (2) Common upregulated genes in neurotoxicity and cardiotoxicity (Supplementary Table S4), (3) Exclusively downregulated genes in neurotoxicity (Supplementary Table S5), (4) Common downregulated genes in neurotoxicity and cardiotoxicity (Supplementary Table S6), 5) Exclusively upregulated genes in cardiotoxicity (Supplementary Table S7), and (6) Exclusively downregulated genes in cardiotoxicity (Supplementary Table S8). Table 2 shows the number of genes in each list. Table 3 shows gene names and their abbreviations. From these genes, only those genes having fold change greater than 3 were considered for induced network modules analysis to introduce stringency in the interaction analysis (Supplementary Tables S3–S8). In the induced network modules of ConsensusPathDB (Humans), “gene set analysis” was used to analyze the six sets of genes. Prior to submission, each gene list was corrected to only include those paralogs of zebrafish that share greater homology to the human ortholog. We employed this approach to find putative gene biomarkers of toxicity that can be extrapolated to humans since majority of pathways are conserved in humans and zebrafish and zebrafish is already an established model of cardiotoxicity and neurotoxicity. Each input gene served as a “seed node” for which we determined, gene regulatory interactions, protein interactions and biochemical interactions in the form of an induced network module. Additionally, the generated networks included genes/proteins etc. called as “intermediate nodes” that associate two or more seed genes with each other and have many significant connections within the entire induced network module. Though these intermediate nodes are associated with the seed nodes, however they did not appear in the input gene list. So, one may speculate that they are not regulated at the transcriptional level in the phenotype under study, but have been otherwise shown in the literature to have some association with the seed nodes. Table 4 shows intermediate node complex genes obtained in our study and their abbreviations.

**Table 2:** Numbers of genes in each list.

List	Total genes	Genes (fold change greater than 3)
Exclusively upregulated genes in neurotoxicity (Supplementary Table S3)	59	19
Commonly upregulated genes in neurotoxicity and cardiotoxicity (Supplementary Table S4)	18	6
Exclusively downregulated genes in neurotoxicity (Supplementary Table S5)	118	40
Common downregulated genes in neurotoxicity and cardiotoxicity (Supplementary Table S6)	10	2
Exclusively upregulated genes in cardiotoxicity (Supplementary Table S7)	14	6
Exclusively downregulated genes in cardiotoxicity (Supplementary Table S8)	49	22

**Table 3:** Gene names and their abbreviations.

Abbreviation	Gene name
IL10	Interleukin-10
LTA	Lymphotoxin alpha
TP53	Tumor suppressor p53
PRKAB1	Protein kinase AMP-activated non-catalytic subunit beta 1
ODC1	Ornithine decarboxylase
CXCL8	C-X-C motif chemokine ligand 8
PLAGL1	Pleiomorphic adenoma gene-like 1
CXCL1	C-X-C motif chemokine ligand 1
NFE2L2	Nuclear factor, erythroid 2 like 2
HMOX1	Heme oxygenase
ARE	Antioxidant response elements
PTGS2	Prostaglandin G/H synthase 2 precursor
ESR1	Estrogen receptor alpha
SOCS3	Suppressor of cytokine signaling 3
PIK3CA	Phosphatidylinositol 3-kinase catalytic subunit, alpha isoform
mTOR	Mammalian target of rapamycin
CDK6	Cyclin-dependent protein kinase 6
ARRB2	Beta-Arrestin 2, isoform 1
CDC42	Cell division cycle 42
EPAS1	Endothelial PAS domain-containing protein 1
SOX9	SRY-Box transcription factor 9
MATN3	Matrilin 3
MATN1	Matrilin 1
MMP9	Matrix metalloproteinase 9
LHCGR	luteinizing hormone/choriogonadotropin receptor
FKBP12	FK506 binding protein 12
CACNA	Voltage gated calcium channel family
GRIA2	Glutamate ionotropic receptor AMPA type subunit 2
GRIA3	Glutamate ionotropic receptor AMPA type subunit 3
FOS	Fos proto-oncogene, AP-1 transcription factor subunit
GPCR	G-protein couple receptor
FLT4	Fms-related tyrosine kinase 4
KDR	Kinase insert domain receptor
VEGFR	Vascular endothelial growth factor receptor
NR4A1	Nuclear receptor 4A subunit 1
STAT	Signal transducers and activators of transcription
ITGB1	Integrin subunit beta 1
MEF2C	Myocyte enhancer factor 2C
NOTCH	Neurogenic locus notch homolog protein
RHO	Rhodopsin
OPN1MW	Opsin 1, medium wave sensitive
OPN1SW	Opsin 1, short wave sensitive
11cRAL	11-cis-retinyl-Green-sensitive opsin, Opsin-1

**Table 4:** Intermediate node complex genes and their abbreviations.

Abbreviation	Gene name
SMAD3	Mothers against decapentaplegic homolog 3
SMAD4	Mothers against decapentaplegic homolog 4
GATA3	GATA binding protein 3
JUN	Jun proto-oncogene, AP-1 transcription factor subunit
YC	MYC proto-oncogene, burkitt lymphoma and high-grade B-cell lymphoma transcription factor
MAX	myc-associated factor X
CBP	CREB binding protein
p300	Histone acetyltransferase p300
HB-EGF	Heparin binding EGF like growth factor
EGFR	Epidermal growth factor receptor
ATF2	Activating transcription factor 2
MacroH2A	Replication-independent histone member of Histone H2A Family
Fra1	Fos-related antigen 1
USF2	Upstream transcription factor 2
NFAT1-c-4	Nuclear factor of activated T cells C4
Jak2	Janus Kinase 2
MIR203	MicroRNA 203
ERK1-2	Extracellular signal-regulated kinase 1–2
ELK1	TS transcription factor ELK1
FIAT	Factor inhibiting ATF4-mediated transcription
FGFR	Fibroblast growth factor receptor
HIF2A	Hypoxia-inducible factor 2-alpha
ARNT	Aryl hydrocarbon receptor nuclear translocator
HHEX	Hematopoietically expressed homeobox
HEY1	Hairy/enhancer-of-split related with YRPW motif protein 1
ETS1	ETS proto-oncogene 1, transcription factor
RelA	Vian reticuloendotheliosis viral oncogene homolog A
p50	Protein 50 (nuclear factor-kappa B)
HDAC7	Histone deacetylase 7
SEMA4A: PLXND1	Semaphorin 4A: Plexin D1
SRF	Serum response factor
MAL	Mal, T cell differentiation protein
SCAI	Suppressor of cancer cell invasion
NKX2-5	NK2 homeobox 5
CSX	Cardiac-specific homebox
FBXW7:SKP:CUL1:RBX1	F-Box And WD repeat domain containing 7
SKP	Skin derived progenitors
CUL1	Cullin 1
RBX1	RING box protein 1

In our analysis, these interactions were determined at the high-confidence option to obtain intermediate nodes. These intermediate nodes were further ranked based on the significance of their association with our supplied gene list given their overall connectivity in the background network. This rank was denoted by a z-score, which is computed using a binomial proportions test and which can be dynamically controlled by the user to create sub-networks with intended stringency^9^. For determining a suitable z-score for our analysis, induced network modules were generated at a z-score threshold of 0, 25, 50, 75, and 100. While the networks generated at z = 0 were too dense to comprehend, networks at z = 100 were too sparse and barely gave any information about gene networks. Thus, z = 75 was chosen as a suitable threshold for generating networks with reliable stringency and sufficient information for identifying important sub-networks regulating the particular phenotype in our study.

## Supplementary Information

Supplementary Tables.

## Data Availability

All data generated or analysed during this study were obtained using ConsensusPathDB and are included in this published article (and its Supplementary Information files).
